# Met/HGF receptor modulates bcl-w expression and inhibits apoptosis in human colorectal cancers

**DOI:** 10.1054/bjoc.2000.1301

**Published:** 2000-08-16

**Authors:** S Kitamura, S Kondo, Y Shinomura, S Kanayama, Y Miyazaki, T Kiyohara, S Hiraoka, Y Matsuzawa

**Affiliations:** Department of Internal Medicine and Molecular Science, Graduate School of Medicine, Osaka University, 2–2 Yamadaoka, Suita, Osaka, 565–0871, Japan

**Keywords:** c-met, bcl-w, apoptosis, colorectal cancers

## Abstract

The *met* proto-oncogene is the tyrosine kinase growth factor receptor for hepatocyte growth factor. In the present study, we investigated the role of met expression on the modulation of apoptosis in colorectal tumours. The gene expressions of c- *met* and the anti-apoptotic *bcl* -2 family, including *bcl* -2, *bcl* -x _L_ and bcl-w, were analysed in human colorectal adenomas and adenocarcinomas by using a quantitative polymerase chain-reaction combined with reverse transcription. In seven of 12 adenomas and seven of 11 carcinomas, the c- *met* gene was overexpressed. The *bcl* -w, *bcl* -2 and *bcl* -x _L_ genes were over-expressed in nine, five and six of 12 adenomas and in five, two and seven of 11 carcinomas, respectively. The c- *met* mRNA level in human colorectal adenomas and carcinomas was correlated with *bcl* -w but not with *bcl* -2 or with *bcl* -x _L_ mRNA level. The administration of c- *met* -antisense oligonucleotides decreased Met protein levels in the LoVo human colon cancer cell line. In the case of c- *met* -antisense-treated cells, apoptotic cell death induced by serum deprivation was more prominent, compared to control or c- *met* -nonsense-treated cells. Treatment with c- *met* -antisense oligonucleotides inhibits the gene expression of *bcl* -w in LoVo cells. On the other hand, the gene expression of *bcl* -2 or *bcl* -x _L_ was not affected by treatment with c- *met* -antisense oligonucleotides. These findings suggest that Met expression modulates apoptosis through *bcl* -w expression in colorectal tumours. © 2000 Cancer Research Campaign


*British Journal of Cancer* (2000) **83**(5), 668–673
© 2000 Cancer Research Campaign
doi: 10.1054/ bjoc.2000.1301, available online at http://www.idealibrary.com on IDEAL®

### Met/HGF receptor modulates bcl-w expression and inhibits apoptosis in human colorectal cancers

**S Kitamura, S Kondo, Y Shinomura, S Kanayama, Y Miyazaki, T Kiyohara, S Hiraoka and Y Matsuzawa**

Department of Internal Medicine and Molecular Science, Graduate School of Medicine, Osaka University, 2–2, Yamadaoka, Suita, Osaka 565–0871, Japan

**Summary** The *met* proto-oncogene is the tyrosine kinase growth factor receptor for hepatocyte growth factor. In the present study, we investigated the role of met expression on the modulation of apoptosis in colorectal tumours. The gene expressions of c-*met* and the anti-apoptotic *bcl*-2 family, including *bcl*-2, *bcl*-$x_L$ and bcl-w, were analysed in human colorectal adenomas and adenocarcinomas by using a quantitative polymerase chain-reaction combined with reverse transcription. In seven of 12 adenomas and seven of 11 carcinomas, the c-*met* gene was overexpressed. The *bcl*-w, *bcl*-2 and *bcl*-$x_L$ genes were over-expressed in nine, five and six of 12 adenomas and in five, two and seven of 11 carcinomas, respectively. The c-*met* mRNA level in human colorectal adenomas and carcinomas was correlated with *bcl*-w but not with *bcl*-2 or with *bcl*-$x_L$ mRNA level. The administration of c-*met*-antisense oligonucleotides decreased Met protein levels in the LoVo human colon cancer cell line. In the case of c-*met*-antisense-treated cells, apoptotic cell death induced by serum deprivation was more prominent, compared to control or c-*met*-nonsense-treated cells. Treatment with c-*met*-antisense oligonucleotides inhibits the gene expression of *bcl*-w in LoVo cells. On the other hand, the gene expression of *bcl*-2 or *bcl*-$x_L$ was not affected by treatment with c-*met*-antisense oligonucleotides. These findings suggest that Met expression modulates apoptosis through *bcl*-w expression in colorectal tumours.
© 2000 Cancer Research Campaign

**Keywords:** c-met; bcl-w; apoptosis; colorectal cancers

The c-*met* oncogene encodes a cell-surface tyrosine kinase receptor for hepatocyte growth factor (HGF)/scatter factor (Bottaro et al, 1991; Naldini et al, 1991), a potent mitogen for epithelial cells which also promotes cell movement. The HGF-Met signaling pathway has been shown to be involved in tumour development and progression. The coexpession of HGF and Met molecules, which generates an autocrine stimulatory loop, is tumourigenic in NIH3T3 cells (Rong et al, 1992). In addition to tumorigenicity, deregulated HGF-Met signalling has also been shown to enhance in vitro invasiveness (Bhargava et al, 1992; Giordano et al, 1993; Rong et al, 1994; Rosen et al, 1994) and in vivo metastatic potential (Rong et al, 1994; Rosen et al, 1994) of various cell types. The results from several recent studies suggest that the HGF-Met system modulates the process of apoptosis. For example, HGF has been shown to protect canine renal tubular epithelial cells against apoptosis induced by the detachment of cells from their substrate (Frisch and Francis, 1994). An infusion of HGF inhibited apoptosis of rat hepatic ductal cells after the administration of the hepatocarcinogen 2-acetylaminofluorene (Nagy et al, 1996). Furthermore, transgenic expression in the liver of truncated Met, a constitutively active oncogenic form of c-*met*, blocked staurosporine-induced apoptosis of hepatocytes (Amicine et al, 1997).

It has been reported that the c-*met* gene is over-expressed in a high percentage of colorectal adenomas and carcinomas (Liu et al, 1992; Di Renzo et al, 1995; Umeki et al, 1999). However, the issue of whether Met expression in colorectal tumours is involved in resistance to apoptosis is presently unclear. The purpose of this study was to investigate the role of Met expression in the modulation of apoptosis in colorectal tumours. In the present study, the gene expressions of c-*met* and the anti-apoptotic *bcl*-2 family, including *bcl*-2, *bcl*-$x_L$ and *bcl*-w, were analysed in human colorectal adenomas and adenocarcinomas by means of a quantitative polymerase chain reaction combined with reverse transcription (RT-PCR), a technique which we have already employed in other studies (Kondo et al, 1995; 1996). An apparent correlation between c-*met* and *bcl*-w mRNA levels in colorectal tumours was found. Furthermore, we showed that the inhibition of Met production by c-*met* antisense promotes apoptosis induced by serum deprivation and inhibits the gene expression of *bcl*-w but not *bcl*-2 or *bcl*-$x_L$ in LoVo human colon cancer cells.

*Received 15 February 2000*
*Revised 26 March 2000*
*Accepted 17 April 2000*

*Correspondence to:* S Kondo

### MATERIALS AND METHODS

### Subjects

The study involved five control subjects (three males and two females; age range, 36–58 years) with no colorectal lesions as evidenced by endoscopy, 12 patients with colorectal adenoma (seven males and five females; age range, 46–77 years) and 11 patients with colorectal carcinoma (seven males and four females; age range, 35–71 years). Informed consent was obtained from each subject. Four tissue specimens were obtained from normal control mucosa, adenomas, carcinomas, and surrounding non-neoplastic mucosa in the case of patients with an adenoma or carcinoma for histologic evaluation and extraction of RNA. Formalin-fixed, paraffin-embedded tumours were stained with haematoxylin and eosin and classified histologically by expert pathologists as well-differentiated adenocarcinoma ($n$ = 10), moderately differentiated adenocarcinoma (*n* = 1), adenoma without severe dysplasia (*n* = 8), or adenoma with severe dysplasia (*n* = 4). This study was approved by ethical committee.

### Cells and cell culture

Human colon cancer LoVo cells were cultured in Ham's F-12 medium (Gibco BRL, Grand Island, NY, USA) supplemented with 10% heat-inactivated fetal calf serum (Whittaker Bioproducts, Walkersville, MD, USA), 100 units $ml^{-1}$ penicillin, and 100 mg $ml^{-1}$ streptomycin in a water-saturated atmosphere containing 5% $CO_2$ at 37°C.

### RNA extraction

Total RNA was extracted from tissue specimens (three of the biopsy specimens) and LoVo cells using the guanidinium thio-cyanate/acid phenol method. As an internal standard, total RNA was extracted from mouse and rat colon.

### Oligonucleotides used for amplification

In order to amplify c-*met*, *bcl*-w, *bcl*-2 and *bcl*-$x_L$ mRNA in tissue specimens and LoVo cells by nested PCR, two pairs of primers were synthesized. The primer sequences were as follows: (a) 5′-GATCTGGGCAGTGAATTAGT-3′ and 5′-TCTT TCATGAT-GATTCCCCTC-3′ for the first-step c-*met* amplification (Park et al, 1987; Chan et al, 1988); (b) 5′-GTAAGTGCCCGAAGTG-TAAG-3′ and 5′-CAAGGATTTCACAGCACAGT-3′ for the second-step c-*met* amplification; (c) 5′-TATAAGCTGAGGCA-GAAGGG-3′ and 5′-TCAGCACTGTCCTCACTGAT-3′ for the first-step *bcl*-w amplification (Gibson et al, 1996); (d) 5′-TTAT-GTCTGTGGAGCTGG CC-3′ and 5′-TCTCCAGGTAGGCCAC-CATC-3′ for the second-step *bcl*-w amplification; (e) 5′-CAGCTGCACCTGACGCCCTT-3′ (which is also used for the second-step *bcl*-2 amplification) and 5′-GCCTCCGTTATCCTG-GATCC-3′ for the first-step *bcl*-2 amplification (Cleary et al, 1986; Negrini et al, 1987); (f) 5′-CAGCT GCACCTGACGC-CCTT-3′ and 5′-GCCGGTTCAGGTACTCAGTC-3′ for the second-step *bcl*-2 amplification; (g) 5′-AAGGATACAGCTG-GAGTCAG-3′ and 5′-ATCCGACTCACCAATACCTG-3′ for the first-step *bcl*-$x_L$ amplification (Boise et al, 1993; González-García et al, 1994); (h) 5′-ATCAATGGCAACCCATCCTG-3′ and 5′-CTACGCTTTCCACGCACAGT-3′ for the second-step *bcl*-$x_L$ amplification. For the amplification of 28S rRNA, a pair of PCR primers was synthesized. The sequences were 5′-CCATGT-GAACAGCAGTTGAA-3′ and 5′-CCCTGCCCTTCACAAA-GAAA-3′ (Hadjiolov et al, 1984; González et al, 1985). Although both the human and internal standard cDNAs originating from c-*met* mRNA, *bcl*-w mRNA, *bcl*-2 mRNA, *bcl*-$x_L$ mRNA, or *28S* rRNA are amplified by the same primers, only the human DNA fragments amplified with these primers contained an, Mva I (c-*met*), Dpn II (*bcl*-w), Ban II (*bcl*-2), Ava I (*bcl*-$x_L$), or Eae I (*28S* rRNA) restriction endonuclease site.

### RT-PCR

The conversion of RNA to cDNA was carried out in a final volume of 5 μl containing 1 μg or less human and mouse RNA mixture of different proportion in the reaction solution of 50 mM Tris-HCl, pH 8.3, 50 mM KCl, 10 mM $MgCl_2$, 3 mM dithiothreitol, 10 μM random primer (Takara Shuzo, Kyoto, Japan), 1 mM each dNTPs, 6 units RNase inhibitor (Pharmacia LKB Biotechnology, Tokyo, Japan), and 1 unit RAV-2 reverse transcriptase (Takara Shuzo). The c-*met*, *bcl*-w, *bcl*-2, and *bcl*-$x_L$ cDNAs (0.5 μl of the reverse transcription product) were amplified by nested PCR with two pairs of primers in PCR buffer, containing 10 mM Tris-HCl, pH 8.3, 50 mM KCl, 1.5 mM $MgCl_2$, 0.001% (w/v) gelatin, 400 μM of each dNTP, 1 μM each of the first-step or second-step 5′ and 3′ primers, and 0.25 units of Thermus aquaticus DNA polymerase (Taq polymerase) (Perkin-Elmer Cetus, Norwalk, CT, USA). Each amplification profile involved denaturation at 94°C for 1 min, primer annealing at 55°C for 2 min, and extension at 72°C for 3 min. The profile was repeated for 30 cycles. After the second-step PCR amplification, 1 μl from each sample was added to 20 μl of fresh PCR buffer containing the second-step 5′ and 3′ primers, and subjected to one additional cycle to prevent heterodimeric DNA formation between the human and mouse PCR products (Kondo et al, 1995). For the determination of the *28S* rRNA concentration, rat *28S* rRNA was used for the internal standard and the cDNA from human and rat *28S* rRNA was amplified for 30 cycles and one additional cycle as described previously (Kondo et al, 1995).

### Quantitative analysis

After the amplification step was completed, the DNA fragments corresponding to the c-*met* mRNA, *bcl*-w mRNA, *bcl*-2 mRNA, *bcl*-$x_L$ mRNA and *28S* rRNA were digested with Mva I (New England Biolabs, Beverly, MA, USA), Dpn II (Takara Shuzo), Ban II (Takara Shuzo), Ava I (New England Biolabs,) and Eae I (Takara Shuzo), respectively. The digested samples were then injected directly into a high-performance liquid chromatography (HPLC) system with a UV-spectrophotometric detector (Model SPD-6A, Shimadzu, Kyoto, Japan) operated at 260 nm. The analytical column was a TSK gel DEAE-NPR (Tosoh, Tokyo, Japan). The digested DNA fragments were chromatographed under a 20 min linear gradient of NaCl from 0.4–0.6 M prepared in 20 mM Tris-HCl, pH 9.0, at a flow rate of 1.0 ml $min^{-1}$. The HPLC elution profile revealed the baseline separation of three DNA fragments. The resulting fragments were identified as two human fragments (c-*met*: 145 base pairs (bp) and 242 bp: *bcl*-w: 109 bp and 208 bp; *bcl*-2: 55 bp and 144 bp; *bcl*-$x_L$: 80 bp and 236 bp; *28S*:93 bp and 148 bp) and an internal standard fragment (c-*met*: 387 bp; *bcl*-w: 317 bp; *bcl*-2: 199 bp; *bcl*-$x_L$: 316 bp; *28S*: 241 bp). For the determination of the c-*met* mRNA, *bcl*-w mRNA, *bcl*-2 mRNA, *bcl*-$x_L$ mRNA and *28S* rRNA levels, the peak-height ratio of the 242 bp to the 387 bp fragment, the 208 bp to the 317 bp fragment, the 144 bp to the 199 bp fragment, the 236 bp to the 316 bp fragment and the 148 bp to the 241 bp fragment were measured, respectively. The coefficient of variation of this assay was 8.5% for c-*met* mRNA, 9.4% for *bcl*-w mRNA, 9.2% for *bcl*-2 mRNA, 7.2% for *bcl*-$x_L$ mRNA and 9.0% for *28S* rRNA. The c-*met*, *bcl*-w, *bcl*-2 and *bcl*-$x_L$ mRNA levels were expressed as a relative c-*met* mRNA/*28S* rRNA ratio, a relative *bcl*-w mRNA/*28S* rRNA ratio, a relative *bcl*-2 mRNA/*28S* rRNA ratio, and a relative *bcl*-$x_L$ mRNA/*28S* rRNA ratio, respectively.

### Antisense oligonucleotides

HPLC-purified 20-*mer* c-*met* antisense oligonucleotides and nonsense (a scramble version) control oligonucleotides were

*© 2000 Cancer Research Campaign* 

purchased from Bex (Tokyo, Japan). The antisense oligonucleotides straddled the predicted translation-initiation site of human c-*met* mRNA. The sequences used were 5′-ACAGCGGGGGCCTTCATTAT-3′ (c-*met*-antisense) and 5′-TCGGCTACAAGCTACGGTTG-3′ (c-*met*-nonsense).

### Western blot analysis

Cells were washed in PBS and lysed in lysis buffer containing 20 mM Tris (pH 8.0), 137 mM NaCl, 10% glycerol, 1% Nonidet P-40, 10 mM EDTA, 100 mM NaF, 1 mM PMSF, 0.25 TIU $ml^{-1}$ of aprotinin, and 10 μg $ml^{-1}$ of leupeptin. Aliquots containing 30 μg of total protein were size-fractionated by sodium dodecyl sulphate-polyacrylamide gel electrophoresis (5–20% gradient gels). Immunoblot analysis was performed as described previously using a rabbit anti-Met antibody (Santa Cruz Biotechnology, Santa Cruz, CA, USA) (Kitamura et al, 1999). Protein concentrations of the homogenates were determined using a bicinchoninic acid protein assay reagent (Pierce, Rockford, IL, USA).

### Number of viable cells and cell viabilities

LoVo cells were seeded at $5 \times 10^4$ cells per 60 mm dish and cultured in Ham's F-12 medium supplemented with 10% heat-inactivated foetal calf serum. After preincubation with 10 μM c-*met*-antisense or c-*met*-nonsense oligonucleotides for 4 days, medium was removed and replaced with serum-deprived medium containing 10 μM c-*met*-antisense or c-*met*-nonsense oligonucleotides. The serum-deprived medium containing oligonucleotides was not changed until measurement of cell viabilities. The number of viable cells was determined by haemocytometry using trypan blue exclusion.

### Measurement of apoptosis by flow cytometry

DNA degradation was assessed by measuring the DNA content of individual cells by flow cytometry. In preparation for flow cytometry, cells were collected after brief trypsinization, washed with PBS, and fixed in 70% cold ethanol. Samples were then treated with RNase, stained with 10 μg $ml^{-1}$ propidium iodine, and analysed using a Becton Dickinson FACScan. Cell-cycle distributions were quantified using Cell-quest software.

### Statistical analysis

Statistical analysis was made using Wilcoxon matched-pairs signed-rank test or Mann – Whitney U test. A level of $P < 0.05$ was accepted as statistically significant. For the measurements of the levels of c-*met* mRNA, *bcl*-w mRNA, *bcl*-2 mRNA, *bcl*-$x_L$ mRNA and *28S* rRNA, each data-point represents the mean of three measurements.

### RESULTS

### Gene expression of c-*met*, *bcl*-w, *bcl*-2 and *bcl*-$x_L$ in human colorectal adenomas and carcinomas

In seven of 12 (58%) adenomas and seven of 11 (64%) carcinomas, the c-*met* gene was found to be overexpressed (Figure 1), when overexpression is defined as an increase of greater than two-fold over the expression in adjacent normal tissue. The *bcl*-w, *bcl*-2 and *bcl*-$x_L$ genes were overexpressed in nine (75%), five (42%) and six (50%) of 12 adenomas and in five (45%), two (18%) and seven (64%) of 11 carcinomas, respectively. The levels of *bcl*-w mRNA in tumours with c-*met*-overexpression were apparently higher compared with the levels in tumours without c-*met*-overexpression (Figure 2). A significant correlation (correlation coefficient (r) = 0.754, $P < 0.0001$) was observed between the *bcl*-w mRNA levels and c-*met* mRNA levels (data not shown). The levels of *bcl*-2 or *bcl*-$x_L$ mRNA were not correlated with the gene expression of c-*met* in colorectal adenomas and carcinomas.

### Evaluation of c-met protein levels by Western blotting

The c-Met protein in LoVo cells was detected as a single polypeptide with a molecular weight of 190 kDa, a finding consistent with previous reports that LoVo cells express constitutively activated Met protein which is uncleaved because of defective post-translational processing (Mondino et al, 1991). When added to cultures of LoVo cells for 4 days, c-*met*-antisense produced concentration-dependent reductions in the steady state levels of Met protein (Figure 3). In contrast, the Met protein level of 10 μM c-*met*-nonsense-treated cells was similar to that of untreated cells.

### Effect of antisense oligonucleotides to c-*met* on apoptosis induced by serum deprivation

After preincubation of LoVo cells with 10 μM c-*met*-antisense or c-*met*-nonsense oligonucleotides for 4 days, the serum was removed from the medium. Pretreatment with antisense oligonucleotides rapidly decreased the number of viable cells after serum deprivation in a time-dependent manner (Figure 4). Nonsense-

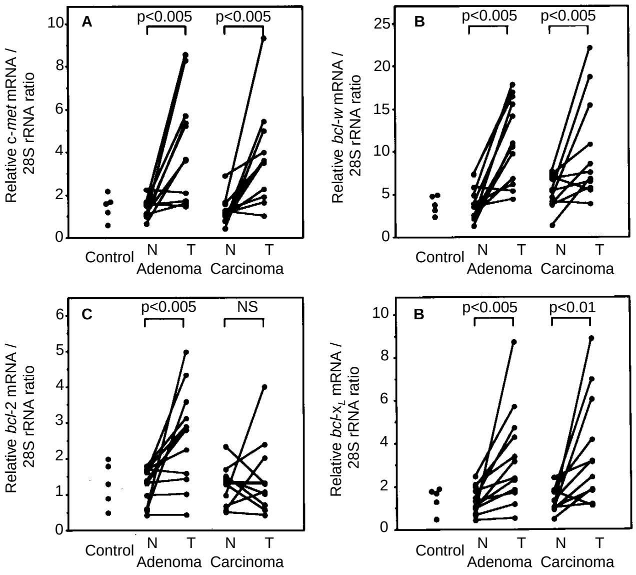

**Figure 1** Gene expression of c-*met* (**A**), *bcl*-w (**B**), *bcl*-2 (**C**) and *bcl*-$x_L$ (**D**) in colorectal adenomas and carcinomas. Tissue specimens were obtained from normal control mucosa, adenomas, carcinomas, and the surrounding non-neoplastic mucosa in patients with an adenoma or carcinoma. The levels of c-*met* mRNA, *bcl*-w mRNA, *bcl*-2 mRNA and *bcl*- $x_L$ mRNA were determined using a quantitative polymerase chain reaction combined with reverse transcription. N and T indicate the surrounding non-neoplastic mucosa and tumour, respectively. Each pair is linked by a solid line. NS; not statistically significant

*© 2000 Cancer Research Campaign*

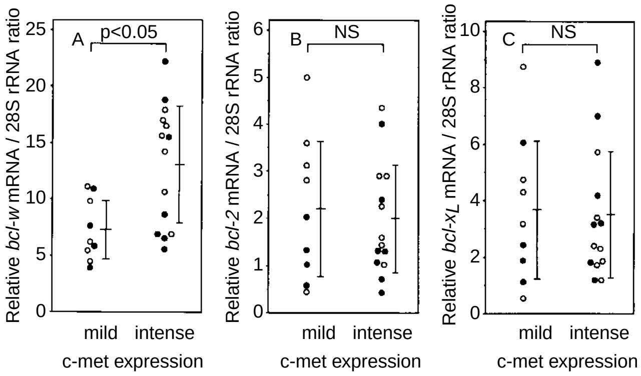

**Figure 2** Correlation between the gene expression of c-*met* and the anti-apoptotic *bcl*-2 family (**A** = *bcl*-w; **B** = *bcl*-2; **C** = *bcl*-$x_L$) in colorectal adenomas (open circle) and carcinomas (closed circle). The degree of c-*met* gene expression relative to the surrounding non-neoplastic mucosa of the same patient are as follows: mild = less than two-fold; intense = an increase of two-fold or more. Vertical bar to the right of each group represents the mean ± standard deviation. NS = not statistically significant

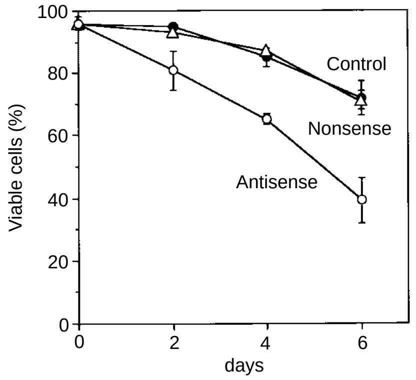

**Figure 4** Effect of antisense oligonucleotides to c-*met* on survival of LoVo cells after serum deprivation. Cells were seeded at 5 × $10^4$ cells per 60 mm dish. After pre-incubation for 4 days with 10 μM c-*met*-antisense (open circle) or c-*met*-nonsense (open triangle) oligonucleotides, serum was removed from the medium. Several cultures received no oligonucleotides (closed circle). Each data-point represents the mean ± standard error (*n* = 4)

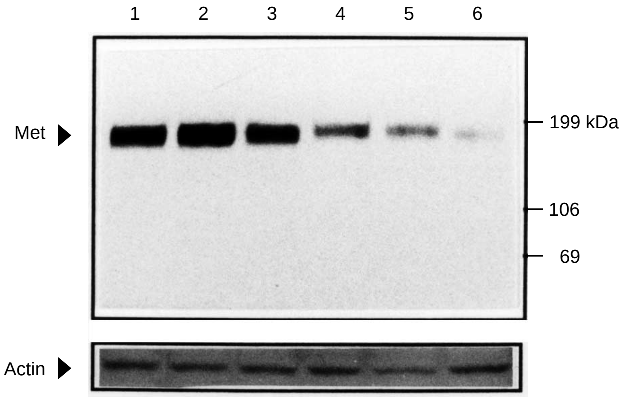

**Figure 3** Western blot analysis of Met protein. Cells were lysed after incubation for 4 days with various concentrations of c-*met*-antisense or c-*met*-nonsense oligonucleotides. Total protein (30 μg) from each sample was loaded on 5–20% gradient SDS-polyacrylamide gels. The samples are: control LoVo cells (lane 1), 10 μM c-met-nonsense (lane 2), 1.25 μM c-*met*-antisense (lane 3), 2.5 μM c-*met*-antisense (lane 4), 5 μM c-*met*-antisense (lane 5), and 10 μM c-*met*-antisense-treated cells (lane 6)

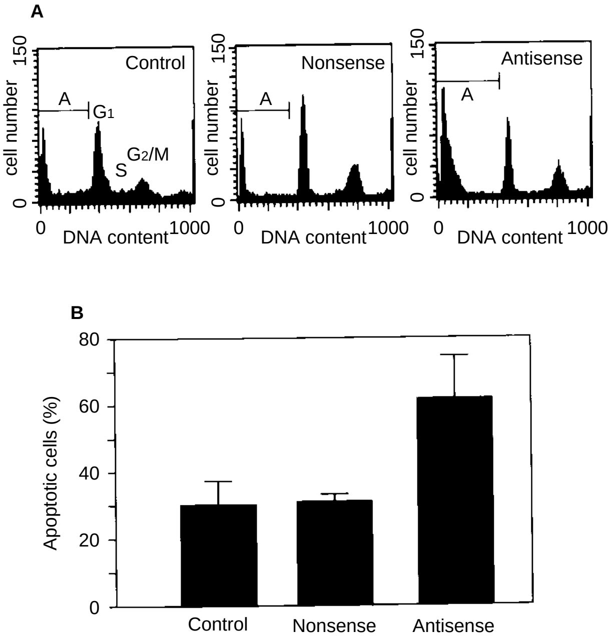

**Figure 5** Effect of c-*met*-antisense oligonucleotides on apoptotic cell death after serum deprivation. LoVo cells were seeded at 5 × $10^4$ cells per 60 mm dish. After pre-incubation for 4 days with 10 μM c-*met*-antisense or c-*met*-nonsense oligonucleotides, the serum was removed from the medium. (**A**) DNA fluorescence histogram of propidium iodide-stained cells after serum deprivation for 6 days. (**B**) The percentage of apoptotic cells after serum deprivation for 6 days. The number of DNA fragmented cells was determined by flow cytometry as the sub-diploid fraction (marked by an 'A' in the histograms) and the percentage of apoptotic cells was calculated. Data are expressed as mean ± standard error (*n* = 4)

treated cells survived for more than 6 days after serum deprivation. In order to determine if the decrease in the number of viable cells after serum deprivation could be attributed to apoptosis, the number of apoptotic cells was measured by flow cytometry. Fragmented DNA contained in apoptotic cells resulted in an unequivocal hypodiploid DNA peak that was distinguishable from the diploid DNA peak seen in LoVo cells after serum deprivation (Figure 5A). The increase in apoptotic cells after serum deprivation was more prominent in c-*met*-antisense-pretreated cells than in the control or c-*met*-nonsense-pretreated cells (Figure 5B).

### Effect of antisense oligonucleotides to c-*met* on gene expression of *bcl*-w, *bcl*-2 and *bcl*-$x_L$

After incubation of LoVo cells with 10 μM c-*met*-antisense or c-*met*-nonsense oligonucleotides for 4 days, expression levels of *bcl*-w, *bcl*-2 and *bcl*-$x_L$ mRNAs were determined by competitive RT-PCR method. In the case of the c-*met*-antisense treated cells, the *bcl*-w mRNA level was decreased when compared to either untreated or c-*met*-nonsense-treated cells (Figure 6). Gene expression of *bcl*-2 or *bcl*-$x_L$ of LoVo cells was not affected by treatment with c-*met*-antisense or c-*met*- nonsense oligonucleotides.

### DISCUSSION

The c-*met* oncogene encodes the receptor for HGF, a potent mitogen for epithelial cells that also promotes cell motility and invasiveness. It has recently been suggested that HGF protects

© 2000 Cancer Research Campaign

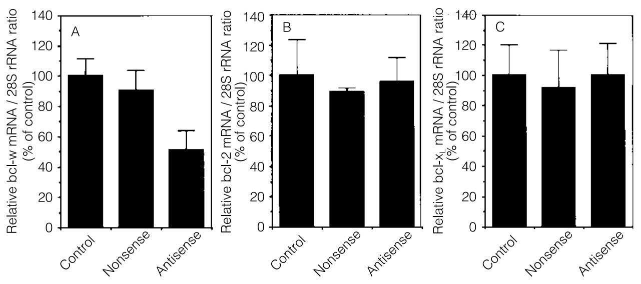

**Figure 6** Effect of c-*met*-antisense oligonucleotides on gene expression of *bcl*-w (**A**), *bcl*-2 (**B**) and *bcl*-$x_L$ (**C**) in LoVo cells. After incubation with 10 μM c-*met*-antisense or c-*met*-nonsense oligonucleotides for 4 days, the expression levels of *bcl*-w, *bcl*-2 and *bcl*-$x_L$ mRNAs were determined by competitive RT-PCR method. Data are expressed as mean ± standard error ($n = 4$)

epithelial cells against apoptosis induced by various stimuli (Frisch and Francis, 1994; Fan et al, 1998). Previous studies have shown that the c-*met* gene is overexpressed in colorectal adenomas and carcinomas (Liu et al, 1992; Di Renzo et al, 1995; Umeki et al, 1999). In the present study, we investigated the role of c-*met* expression in colorectal tumours in the modulation of apoptosis. The relationship between the mRNA expression level of c-*met* and those of the anti-apoptotic *bcl*-2 family including *bcl*-w, *bcl*-2, *bcl*-$x_L$ was examined in colorectal adenomas and carcinomas. The overexpression of the c-*met* gene was detectable in more than 50% of the lesions at both stages of adenomas and carcinomas. Our observations are in agreement with previous reports (Liu et al, 1992; Di Renzo et al, 1995). Up-regulation of the *bcl*-$x_L$ gene was also observed in adenomas and carcinomas. In contrast to *bcl*-$x_L$ gene expression, the gene expression of *bcl*-2 seemed to decline during the progression of adenomas to carcinomas. In some colorectal carcinomas, the expression of *bcl*-2 gene was decreased compared with that in adjacent normal tissues. These results are in agreement with earlier immunohistochemical study in which Bcl-2 and Bcl-$x_L$ protein expression was examined (Krajewska et al, 1996). In this study, we demonstrated, for the first time, the over-expression of the *bcl*-w gene in many colorectal adenomas and carcinomas. Furthermore, the expression level of the *bcl*-w gene was correlated with the c-*met* expression level.

In order to clarify the role of Met expression on cell survival and gene expression of the anti-apoptotic *bcl*-2-related gene, we treated LoVo human colon cancer cells with c-*met*-antisense oligonucleotides. In LoVo cells, although no mutations were present in the Met-coding region, the tyrosine kinase encoded by the c-*met* proto-oncogene is constitutively activated via a defective post-translational processing of the precursor protein (Mondino et al, 1991). c-*met*-antisense oligonucleotides effectively inhibited Met protein production and increased the level of apoptotic cell death after serum deprivation, suggesting that Met expression in colon cancer cells plays an important role in preventing apoptosis. Furthermore, as shown in Figure 6, the inhibition of Met production resulted in a decrease in gene expression of *bcl*-w but not *bcl*-2 or *bcl*-$x_L$ in LoVo cells.

*bcl*-w has been cloned as a *bcl*-2-related gene and has been shown to be expressed in many tissues, especially the colon (Gibson et al, 1996). It has been shown that the enforced expression of *bcl*-w enhances survival of several cell types exposed to a variety of cytotoxic agents. High levels of Bcl-w enables a myeloid cell line to resist apoptosis induced by IL-3 withdrawal or γ-irradiation and a T hybridoma cell line is rendered refractory to γ-irradiation and dexamathasone (Gibson et al, 1996). These facts, along with our results that the decrease in Met protein content inhibited *bcl*-w expression and resulted in increased cell death induced by serum deprivation, suggest that Met enhances cell survival through the modulation of Bcl-w expression.

In a recent study using MDA-MB-453 human breast cancer cells, it has been reported that pretreatment of MDA-MB-453 cells with HGF inhibited the adriamycin-induced decrease in the levels of Bcl-$x_L$ (Fan et al, 1998), suggesting that Bcl-$x_L$ may have a role in HGF-mediated cell survival in some cancer cells. However, in colorectal tumours, no relationship between c-*met* and *bcl*-$x_L$ gene expression was observed. Furthermore, the inhibition of Met protein production by c-*met*-antisense had no effect on the expression level of *bcl*-$x_L$ gene.

In conclusion, we demonstrated that the expression of c-*met* gene is up-regulated and correlated with *bcl*-w expression in human colorectal adenomas and carcinomas. The inhibition of Met protein expression resulted in a decrease in *bcl*-w gene expression and an increase in apoptotic cell death induced by serum deprivation in LoVo human colon cancer cells. These observations suggest that Met expression plays a role in the protection of cells against apoptosis by targeting Bcl-w in colorectal tumours.

### REFERENCES

Amicine L, Spagnoli FM, Späth G, Giordano S, Tommasini C, Bernardini S, De Luca V, Della Rocca C, Weiss MC, Comoglio PM and Tripodi M (1997) Transgenic expression in the liver of truncated Met blocks apoptosis and permits immortalization of hepatocytes. *EMBO J* **16**: 495–503

Bhargava M, Knesel JJ, Halaban R, Li Y, Pang S, Goldberg I, Setter E, Donovan MA, Zaraegar R, Michalopoulos GA, Nakamura T, Falleto D and Rosen EM (1992) Scatter factor and hepatocyte growth factor: activities, properties and mechanisms. *Cell Growth Differ* **3**: 11–20

Boise LH, González-Garcia M, Postema CE, Ding L, Lindsten T, Turka LA, Mao X, Nunez G and Thompson CB (1993) bcl-x, a bcl-2-related gene that functions as a dominant regulator of apoptotic cell death. *Cell* **74**: 597–608

Bottaro DP, Rubin JS, Faletto DL, Chan AML, Kmiecik TE, Vande Woude GF and Aaronson SA (1991) The hepatocyte growth factor receptor is the c-met oncogene product. *Science* **251**: 802–804

Chan AM, King HW, Deakin EA, Tempest PR, Hilkens J, Kroezen V, Edwards DR, Wills AJ, Brookes P and Cooper CS (1988) Characterization of the mouse met proto-oncogene. *Oncogene* **2**: 593–599

Cleary ML, Smith SD and Sklar J (1986) Cloning and structural analysis of cDNAs for bcl-2 and a hybrid bcl-2/immunoglobulin transcript resulting from the t(14;18) translocation. *Cell* **47**: 19–28

Di Renzo MF, Olivero M, Giacomini A, Porte H, Chastre E, Mirossay L, Nordlinger B, Bretti S, Bottardi S, Giordano S, Plebani M, Gespach C and Comoglio PM (1995) Overexpression and amplification of the Met/HGF receptor gene during the progression of colorectal cancer. *Clin Cancer Res* **1**: 147–154

Fan S, Wang J-A, Yuan R-Q, Rockwell S, Janet A, Zlatapolskiy A, Goldberg ID and Rosen EM (1998) Scatter factor protects epithelial and carcinoma cells against apoptosis induced by DNA-damaging agents. *Oncogene* **17**: 131–141

Frisch SM and Francis H (1994) Disruption of epithelial cell-matrix interactions induces apoptosis. *J Cell Biol* **124**: 619–626

Gibson L, Holmgreen SP, Huang DCS, Bernard O, Copeland NG, Jenkins NA, Sutherland GR, Baker E, Adams JM and Cory S (1996) bcl-w, a novel member of the bcl-2 family, promotes cell survival. *Oncogene* **13**: 665–675

Giordano S, Zhen Z, Medico E, Gaudino G, Galimi F and Comoglio PM (1993) Transfer of motogenic and invasive response to scatter factor/hepatocyte growth factor by transfection of human MET protooncogene. *Proc Natl Acad Sci USA* **90**: 649–653

González-García M, Pérez-Ballestero R, Ding L, Duan L, Boise LH, Thompson CB and Núnez G (1994) bcl-$x_L$ is the major bcl-x mRNA from expressed during murine development and its product localizes to mitochondria. *Development* **120**: 3033–3042

 © 2000 Cancer Research Campaign

González IL Gorski JL Campen TJ, Dorney DJ, Erickson JM, Sylvester JE and Schmickel RD (1985) Variation among human 28S ribosomal RNA gene. *Proc Natl Acad Sci USA* **82**: 7666–7670

Hadjiolov AA, Georgiev OI, Nosikov VV and Yavachev LP (1984) Primary and secondary structure of rat 28S ribosomal RNA. *Nucleic Acid Res* **25**: 3677–3693

Kitamura S, Miyazaki Y, Shinomura Y, Kondo S, Kanayama S and Matsuzawa Y (1999) Peroxisome proliferator-activated receptor γ induces growth arrest and differentiation markers of human colon cancer cells. *Jpn J Cancer Res* **90**: 75–80

Kondo S, Shinomura Y, Kanayama S, Higashimoto Y, Kiyohara T, Yasunaga Y, Kitamura S, Ueyama H, Imamura I, Fukui H and Matsuzawa Y (1995) Helicobacter pylori increases gene expression of hepatocyte growth factor in human gastric mucosa. *Biochem Biophys Res Commun* **210**: 960–965

Kondo S, Shinomura Y, Kanayama S, Higashimoto Y, Miyagawa J-I, Minami T, Kiyohara T, Zushi S, Kitamura S, Isozaki K and Matsuzawa Y (1996) Overexpression of bcl-$x_L$ gene in human gastric adenomas and carcinomas. *Int J Cancer* **68**: 727–730

Krajewska M, Moss SF, Krajewski S, Song K, Holt PR and Reed JC (1996) Elevated Expression of Bcl-x and reduced Bak in primary colorectal adenocarcinomas. *Cancer Res* **56**: 2422–2427

Liu C, Park M and Tsao M-S (1992) Overexpression of c-met proto-oncogene but not epidermal growth factor receptor pr c-erbB-2 in primary human colorectal carcinomas. *Oncogene* **7**: 181–185

Mondino A, Giordano S and Comoglio PM (1991) Defective posttranslational processing activates the tyrosine kinase encoded by the Met proto-oncogene (hepatocyte growth factor receptor). *Mol Cell Biol* **11**: 6084–6092

Nagy P, Bisgaard HC, Santoni-Rugiu E and Thorgeirsson SS (1996) In vivo infusion of growth factors enhances the mitogenic response of rat hepatic ductal (oval) cells after administration of 2-acetylaminofluorene. *Hepatology* **23**: 71–79

Naldini L, Vigna E, Narsimhan RP, Gandino G, Zarnegar R, Michalopoulos GK and Comoglio PM (1991) Hepatocyte growth factor (HGF) stimulates the tyrosine kinase activity of the receptor encoded by the proto-oncogene c-met. *Oncogene* **6**: 501–504

Negrini M, Silini E, Kozak CA, Tsujimoto Y and Croce CM (1987) Molecular analysis of mbcl-2: structure and expression of the murine gene homologous to the human gene involved in follicular lymphoma. *Cell* **49**: 455–463

Park M, Dean M, Kaul K, Braun MJ, Gonda MA and Vande Woude G (1987) Sequence of MET protooncogene cDNA has features characteristic of the tyrosine kinase family of growth-factor receptors. *Proc Natl Acad Sci USA* **84**: 6379–6383

Rong S, Bodescot M, Blair D, Nakamura T, Mizuno K, Park M, Chan A, Aaronson S and Vande Woude GF (1992) Tumorigenicity of the met proto-oncogene and the gene for hepatocyte growth factor. *Mol Cell Biol* **12**: 5152–5158

Rong S, Segal S, Anver M, Resau JH and Vande Woude GF (1994) Invasiveness and metastasis of NIH 3T3 cells induced by Met-hepatocyte growth factor/scatter factor autocrine stimulation. *Proc Natl Acad Sci USA* **99**: 4731–4735

Rosen EM, Knesel J, Goldberg ID, Bhargava M, Joseph A, Zitnik R, Wines J, Kelley M and Rockwell S (1994) Scatter factor modulates the metastatic phenotype of the EMT6 mouse mammary tumor. *Int J Cancer* **57**: 706–714

Umeki K, Shiota G and Kawasaki H (1999) Clinical significance of c-met oncogene alteration in human colorectal cancer. *Oncology* **56**: 314–321

*© 2000 Cancer Research Campaign*
